# Dual Stimuli-Triggered Nanogels in Response to Temperature and pH Changes for Controlled Drug Release

**DOI:** 10.1186/s11671-019-2909-y

**Published:** 2019-03-04

**Authors:** Yun Kyoung Kim, Eun-Joong Kim, Jae Hyun Lim, Heui Kyoung Cho, Woo Jin Hong, Hyang Hwa Jeon, Bong Geun Chung

**Affiliations:** 1 Department of Biomedical Engineering, Sogang University, Seoul, 04107 South Korea; 2 Research Center, Sogang University, Seoul, 04107 South Korea; 3 Cosmetic Research Center, Coway Co. Ltd., Seoul, 08502 South Korea; 4 Department of Mechanical Engineering, Sogang University, Seoul, 04107 South Korea

**Keywords:** PNIPAM, LCST, Controlled drug release, Temperature, pH

## Abstract

Poly-*N*-isopropyl acrylamide (PNIPAM) nanogels have been modified with different acrylic acid (AAc) contents for the efficient control of lower critical solution temperature (LCST). In this study, PNIPAM-co-AAc nanogels nanogels showed two volume phase transitions in comparison with PNIPAM. The transition temperature of PNIPAM nanogels was increased with AAc contents. The controlled drug release performance of PNIPAM-co-AAc nanogels loaded with β-lapachone was attributed to the AAc content ratio and was efficiently triggered in response to temperature and pH. Moreover, a colorimetric cell proliferation assay and direct fluorescence-based live/dead staining were used to confirm the concurrence on drug release profiles. Finally, PNIPAM-co-AAc20 showed a relatively low level of drug release in the range of acidic to neutral pH at body temperature, while maximizing drug release at basic pH. Therefore, we demonstrated that the PNIPAM-based nanogel with the temperature- and pH-responsive features could be a promising nanocarrier for potential intestine-specific drug delivery.

**Electronic supplementary material:**

The online version of this article (10.1186/s11671-019-2909-y) contains supplementary material, which is available to authorized users.

## Introduction

Stimuli-responsive nanocarriers have generally been developed as drug delivery systems for therapy, imaging, and diagnostics [1, 2]. Recently, various stimuli including pH, temperature, biomolecules, redox, magnetic field, and ultraviolet light have been used to induce sustained or controlled drug release via an internal or external activation [3–6]. Among these stimuli, pH and temperature are the most well-known modalities in drug delivery and release systems. Poly-*N*-isopropyl acrylamide (PNIPAM) is a representative temperature-responsive polymer that has been utilized in drug reservoirs and release systems. This thermo-sensitive polymer has the ability to alter its phase behavior, exhibiting a swollen state because of hydrogen bonding between water and amide functional groups at the lower critical solution temperature (LCST) and conversely exhibiting shrinkage of the polymer network via hydrophobic interactions above the LCST [7–9]. Moreover, LCST can be commonly controlled by the complexing ratio of acrylic acid (AAc) or acrylic amide coupled with PNIPAM [10, 11]. Specifically, AAc can make two phase transitions when LCST is shifted to higher temperatures [12, 13]. PNIPAM-co-AAc nanogels start to shrink above the LCST due to hydrophobic interactions [14, 15]. However, deprotonation of carboxylic groups in AAc causes an increase in nanogel diameter because of the interelectronic repulsion and the increased osmotic pressure [16–18].

PNIPAM-mediated drug delivery systems have been developed for various applications in biomedical fields. Temperature- or pH-sensitive PNIPAM nanogels have been used to optimize the process of drug adsorption and delivery due to the reversible phase transition property [19–22]. In particular, it has been reported that pH values in different tissues are considered for oral delivery, although there are more subtle changes within different tissues [23–26]. To date, the intelligent biomaterials that can generate a cooperative response under multiple stimuli, such as pH and temperature, have shown advantages over those systems sensitive to a single stimulus [27–29]. The change in hydrophilicity induced by the temperature sensitivity, which can be tailored to occur spontaneously at environmental pH, may also play an important role in pH-sensitivity along with the LCST behavior of the co-polymers and gels.

β-lapachone (β-LP), a natural compound, showed the therapeutic activity in cancer treatment [30]. In biomedicine, the functionalized carriers of β-LP have been designed with the aim of minimizing its toxic effects. Various carriers for β-LP delivery have been developed using gold, graphene oxide, and PNIPAM [31, 32]. To date, β-LP-loaded PNIPAM has been applied to chemotherapeutic regimens in liver, breast, prostate, and colon cancers [33–36]. Although several β-LP carriers have been studied, the relatively complex preparation procedures was uncontrolled or spontaneous β-LP release partly restrained their efficiency. Thus, developing efficient carriers of β-LP for biomedical applications still remains an important task.

**Scheme 1:**
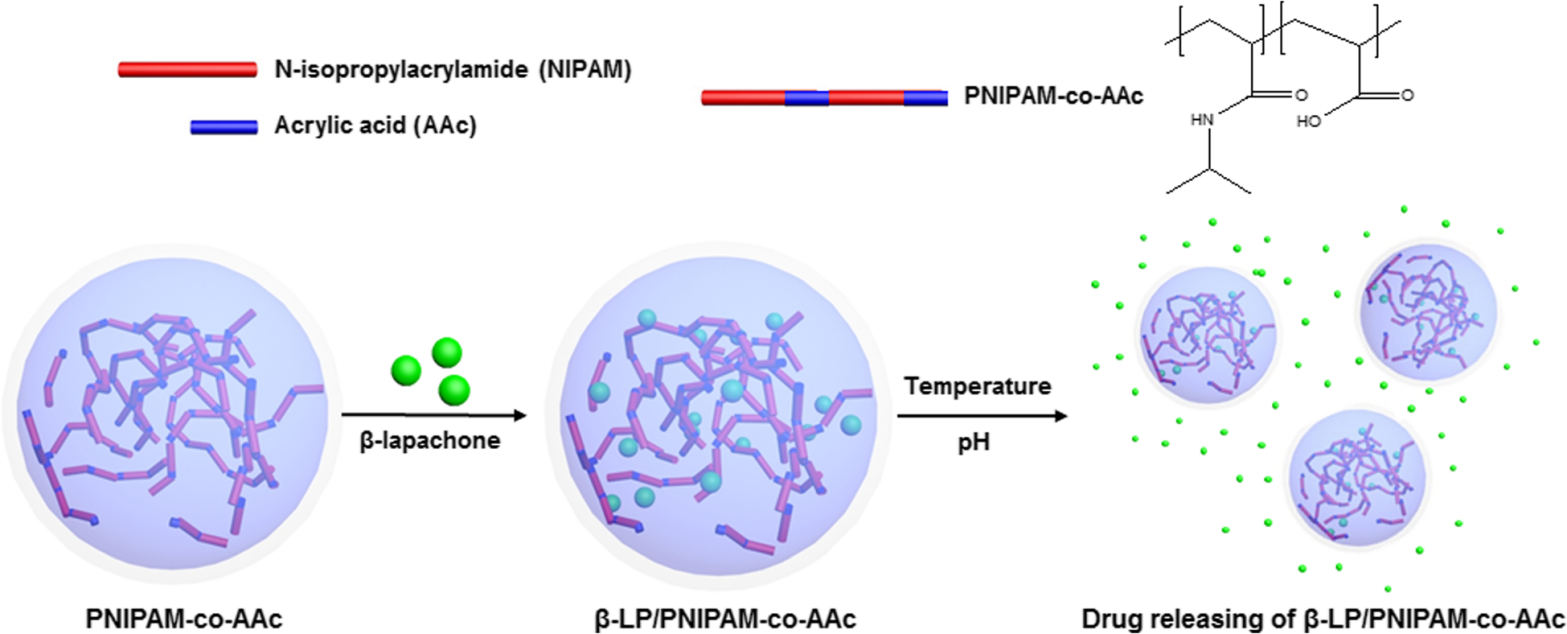
Schematic illustration of double controlled drug release of PNIPAM-co-AAc hydrogels via temperature and pH

Herein, we developed a bi-directional controlled release system using the thermo- and pH-sensitive properties of PNIPAM. This drug delivery system consists of PNIPAM nanogel co-polymerized with AAc contents forming a PNIPAM-co-AAc nanogel. We described a schematic representation of the self-assembly strategy, drug loading, and release of PNIPAM-co-AAc nanogel (Scheme 1). β-LP, a model drug, was loaded into PNIPAM-co-AAc nanogels via hydrophobic interactions. The release of β-LP by the loaded PNIPAM-co-AAc nanogels could be effectively controlled by temperature and pH. PNIPAM-co-AAc nanogels showed an effective anti-proliferative property in fibroblasts with basic pH at body temperature. β-LP loaded in nanogels achieved significant therapeutic efficacy with a thermo- and pH-responsive structure, hence PNIPAM-modified nanogel could be a good candidate for stimuli-responsive drug delivery and treatment of tumors.

## Methods

### Materials

NIPAM (97%, Sigma-Aldrich, St. Louis, MO, USA) was dried under vacuum at room temperature. *N*,*N*′-methylenebisacrylamide (MBA), AAc, distilled water, ethyl alcohol (EtOH), potassium persulfate (KPS) (98%, Dae Jung, KOREA), β-LP (Natural Products, Korea), and phosphate-buffered saline (PBS) were all of analytical grade and used without further purification.

### Synthesis of the PNIPAM-co-AAc Nanogel

PNIPAM-co-AAc nanogel was synthesized according to previous reports [37]. In a 500 mL three-neck round-bottom flask, 2.26 g of NIPAM monomer, 0.154 g MBA as a crosslinking agent, and 0 g, 0.036 g, 0.077 g, 0.145 g of AAc were added into 200 mL distilled water and then dissolved by stirring with a magnetic bar for 30 min at 75 °C, followed by the synthesis of PNIPAM, PNIPAM-co-AAc5, PNIPAM-co-AAc10, and PNIPAM-co-AAc20, respectively. Oxygen was removed from the mixture by nitrogen purging. To initiate the reaction, 37.5 mg KPS as an initiator was added to the solution and then stirred. A reflux condenser was used to prevent the evaporation of the solution due to the high temperature. The solution became turbid within 10 min after the addition of KPS. To remove unreacted monomers, it was dialyzed with a dialysis tube (12–14 kDa) for 7 days. The distilled water used for dialysis was changed daily. The obtained materials were frozen in liquid nitrogen and lyophilized for 3 days to obtain dried PNIPAM-co-AAc nanogel.

### β-LP Loading into PNIPAM-co-AAc

One milligram of the synthesized PNIPAM-co-AAc nanogel was dissolved in 1 mL ethanol, and 0.1 mg β-LP was added to the dissolved PNIPAM-co-AAc. The mixture was vigorously stirred at room temperature in the dark overnight. After stirring, the unencapsulated β-LP was dialyzed with a dialysis tube (6–8 kDa). The dialyzed nanogel was frozen in liquid nitrogen and lyophilized for 3 days. Then, 1 mL of PNIPAM-co-AAc-encapsulated β-LP was injected into the dialysis tube (6–8 kDa). To prevent the loss of solution, the end of the tube was sealed. After adding 10 mL ethanol, the prepared dialysis tubes were immersed in PBS solution.

### Characterization of PNIPAM-co-AAc

Morphology was determined by transmission electron microscopy (TEM) and field emission scanning electron microscopy (FE-SEM). Briefly, after PNIPAM-co-AAc nanogels were sufficiently dispersed using sonication, the dispersions are dropped onto 300 mesh copper grids (Electron Microscopy Science, PA, USA) and evaporated overnight. Then, TEM images were obtained at an accelerating voltage of 200 kV (JEM2100F, JEOL Ltd., Japan). SEM micrographs were scanned at an electron acceleration voltage of 15 kV (JSM-7100F, JEOL USA). The spectra were collected from Fourier-transform infrared spectrometer (FT-IR, Nicolet 6700, Japan). β-LP loading and amount released from the nanogels were calculated by a UV–Vis spectrometer (UV-1800, Shimadzu, Japan). To confirm the LCST, the nanogel was precisely measured at intervals of 1 °C for changes in the size and surface charge of the nanogels using dynamic light scattering (DLS) (ELS-2000ZS, Otsuka Electronics, Japan).

### Drug Release Properties of PNIPAM-co-AAc

To study the release behavior of β-LP, 10 mL of β-LP-loaded nanogels was transferred into a dialysis tube (3.5 kDa), which was then stirred at room temperature and 37 °C in PBS. At a defined release time (0–12 h), 2 mL of the sample in each mixture solution was analyzed by the UV–Vis spectrometer. In the UV–Vis spectrometer, the baseline was set at 200–800 nm with PBS at pH 2, 4, 7.4, and 8, and 2 mL of the released β-LP contained in PBS solution was added to the cuvette.

### Drug Releasing Activity Through Temperature and pH Stimuli

The dual effect on cell viability was evaluated by the 3-(4,5-dimethylthiazol-2-yl)-2,5-diphenyl tetrazolium bromide (MTT) assay. NIH3T3 fibroblast cells were seeded into 96-well plates (2 × 10^4^ cells/well) and cultured overnight at 37 °C. The medium was then replaced by fresh medium containing free β-LP, PNIPAM-co-AAc5, and PNIPAM-co-AAc20 including β-LP at various concentrations. After incubation for 3 h, MTT solution was added into each well and incubated for 4 h. Then, the culture medium was removed, followed by treatment with the solubilization solution. The absorbance values at 595 nm were measured with a microplate reader (EL800, Bio-Tek Instruments, Winooski, VT, USA). The live/dead fluorescence images were captured by a fluorescence microscope (IX37, Olympus, Japan). NIH3T3 cells (1.5 × 10^5^ cells/well) were seeded in μ-Slide 8-well (ibidi, Munich, Germany) and cultured overnight. After replacing the culture medium, 20 μg/mL free β-lapachone, PNIPAM-co-AAc5, and PNIPAM-co-AAc20 including β-LP dispersed in the culture medium was added into the wells. After incubation for 3 h or 6 h, the cells were washed, and cell viability was evaluated by the LIVE/DEAD® Viability/Cytotoxicity Assay (Molecular Probes, Eugene, OR).

## Results and Discussion

### Preparation of PNIPAM-co-AAc Nanogels

**Fig. 1:**
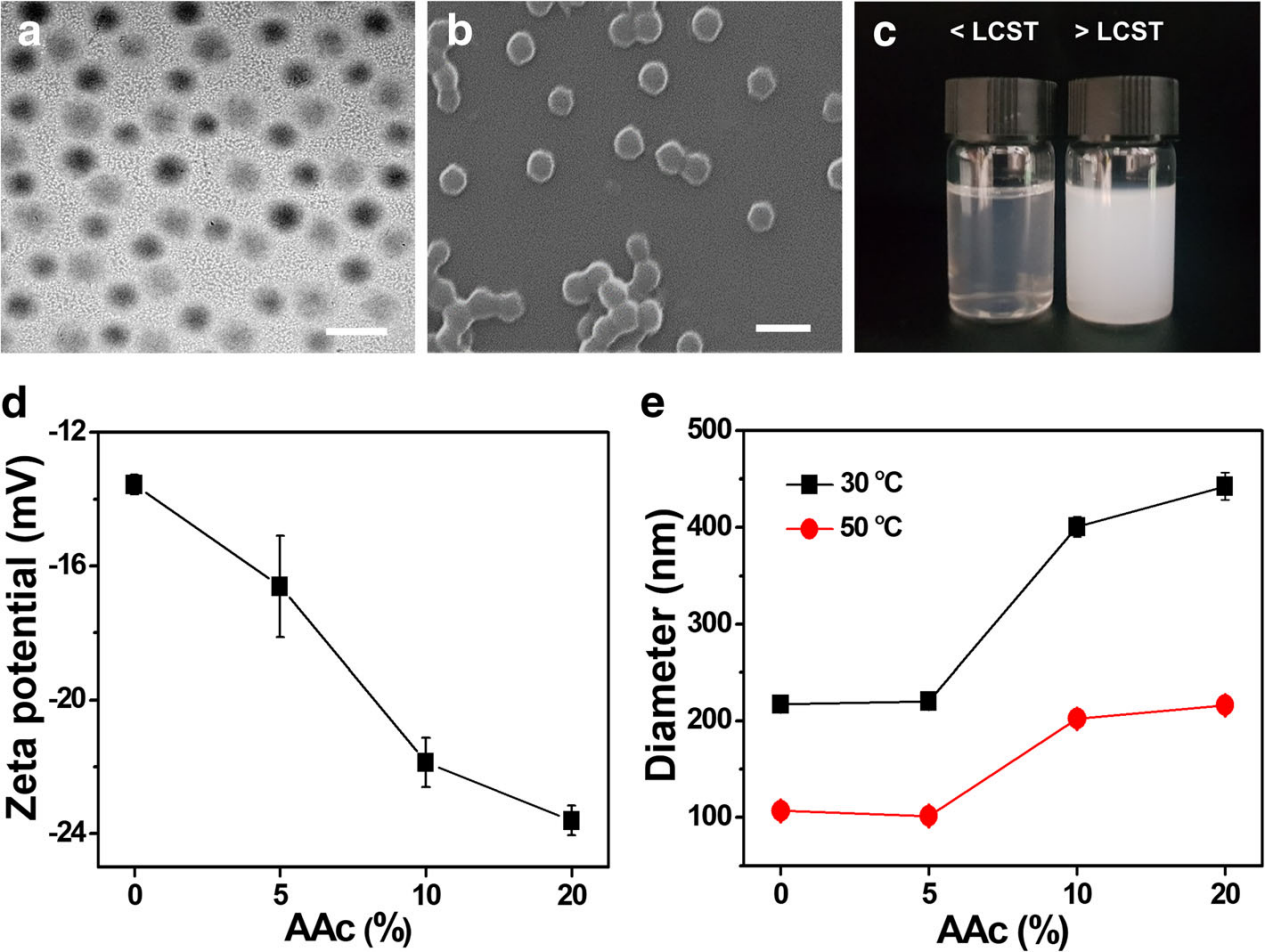
**a** TEM and **b** SEM image of PNIPAM-co-AAc5 nanogels. **c** Physical appearance of PNIPAM-co-AAc5 nanogels. Scale bars are 500 nm. **d** Zeta potentials and **e** average diameters measured at 30 °C and 50 °C by DLS for PNIPAM with 0%, 5%, 10%, and 20% AAc contents at pH 7.4

**Fig. 2:**
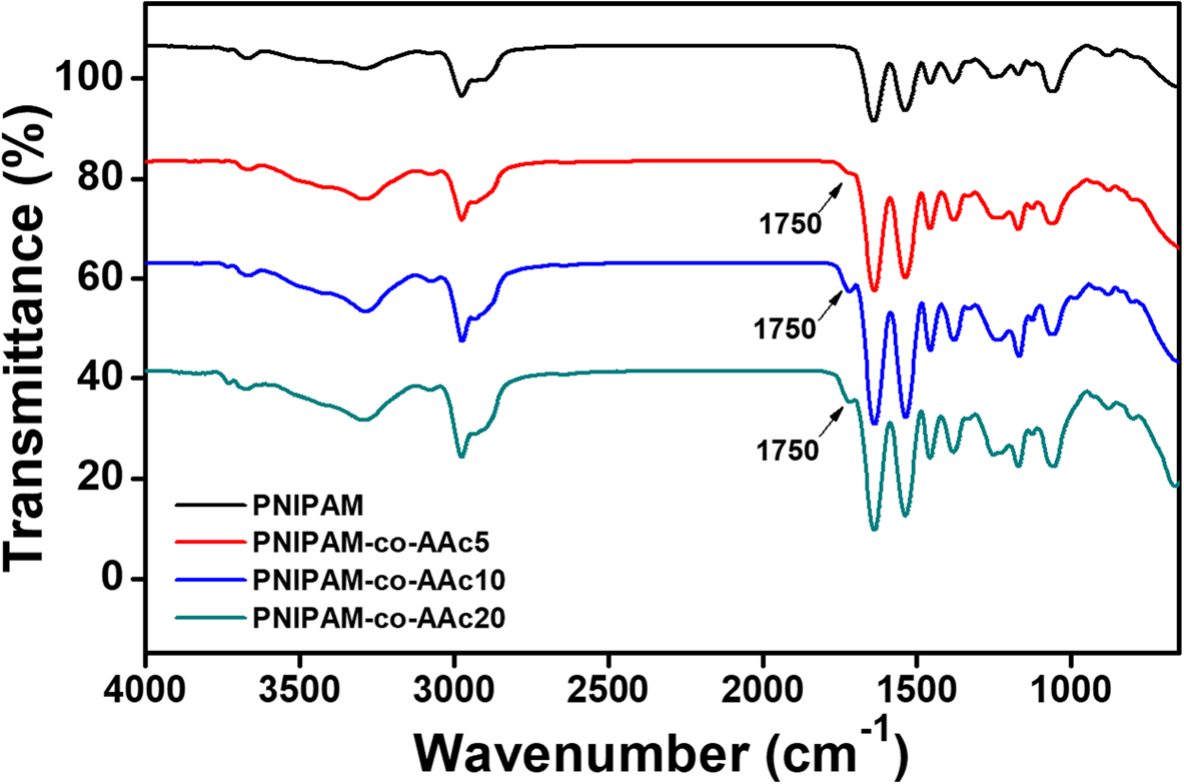
FT-IR spectra of PNIPAM with 0%, 5%, 10%, and 20% AAc contents

PNIPAM-co-AAc nanogels with three different contents of AAc (5, 10, and 20%) were fabricated by a radical polymerization method. TEM and SEM were used to confirm the particle size, morphology, and monodispersity of the nanogels. As shown in Fig. 1a and b, PNIPAM-co-AAc5 nanogel exhibited a relatively uniform size distribution with a mean particle diameter of approximately 250 nm. In addition, the sol-gel transition of the PNIPAM-based nanogels was observed as the temperature increased. Although aqueous solutions of PNIPAM-co-AAc5 persisted as a sol phase at room temperature, the nanogel transitioned into the gel phase upon heating, resulting in the solution becoming turbid above the LCST (Fig. 1c). The zeta potentials of the PNIPAM, PNIPAM-co-AAc5, PNIPAM-co-AAc10, and PNIPAM-co-AAc20 decreased to − 13.56 mV, − 16.61 mV, − 21.87 mV, and − 23.62 mV due to the increased amount of surface carboxyl groups provided by the AAc contents (Fig. 1d). It also indicated that the hydrodynamic diameter of PNIPAM-co-AAc exhibited the range of 217–442 nm as the contents of AAc increased to 30 °C because of increasing hydrogen bonding with water and interelectronic repulsion. However, the nanogel diameters decreased at 50 °C because of hydrophobic interactions (Fig. 1e). These results suggested that PNIPAM-co-AAc can vary in size depending on the amount of AAc linked to PNIPAM and temperature. The composition of the nanogel was further characterized by FT-IR spectroscopy, as shown in Fig. 2. The 1100 cm^−1^~1200 cm^−1^ peak indicated C-N bending. The spectra also displayed the -CH_2_ stretching vibration peak at 1300 cm^−1^~1400 cm^−1^. The additional peak at 1600 cm^−1^~1700 cm^−1^ was attributed to C=O, which belongs to NIPAM. Specifically, carboxylic acid (−COOH) stretching appeared at 1700 cm^−1^~1800 cm^−1^ except for the PNIPAM nanogel. A broad peak at 3200 cm^−1^~3300 cm^−1^ showed the absorption of N-H stretching. Therefore, PNIPAM nanogel derivatives composed of various mixing ratios of PNIPAM and AAc have different characteristics due to the different AAc contents.

### Temperature-Responsive Characteristics

**Fig. 3:**
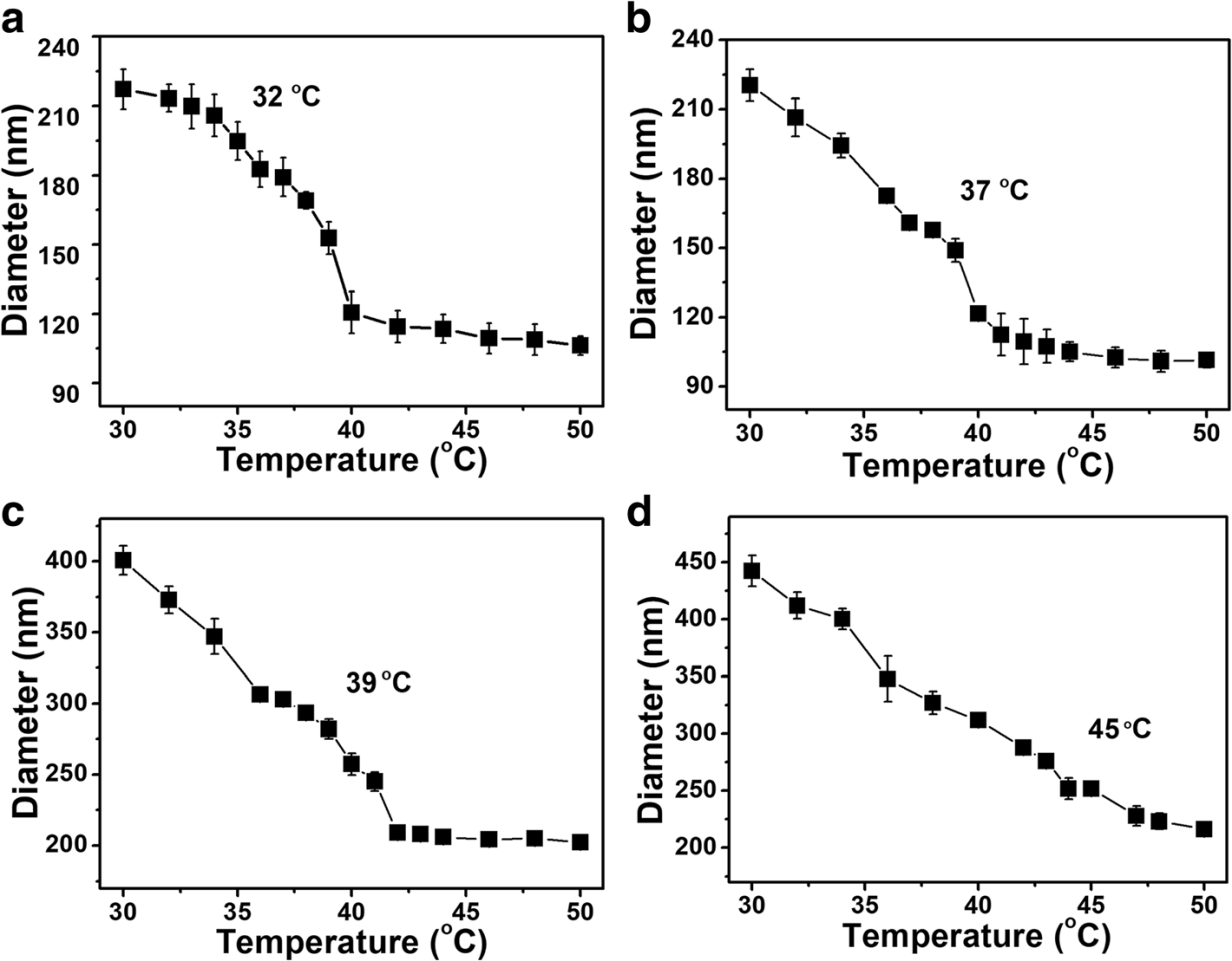
Temperature dependence of the hydrodynamic diameters of **a** PNIPAM, **b** PNIPAM-co-AAc5, **c** PNIPAM-co-AAc10, and **d** PNIPAM-co-AAc20 nanogels at pH 7.4

To investigate the temperature behavior, the size distribution of PNIPAM-co-AAc nanogels was assessed by DLS. The change in the hydrodynamic diameter was measured in the temperature range from 30 to 50 °C to determine the LCST. PNIPAM with 5%, 10%, and 20% AAc contents had two distinct transition steps (Fig. 3). PNIPAM-co-AAc derivatives started the first transition step at 30 °C and then entered the second transition step around 40 °C. Moreover, the second transition temperature tended to increase with increasing AAc contents of the PNIPAM. Therefore, the LCST of PNIPAM-co-AAc20 was at a relatively high temperature of 45 °C, while that of PNIPAM was at 32 °C. This difference in LCST values could be induced by the increased negative charge of PNIPAM-co-AAc derivatives. However, the LCST temperatures of PNIPAM-co-AAc5 and PNIPAM-co-AAc10 were almost identical at 37 °C and 39 °C, respectively. Therefore, PNIPAM-co-AAc10 was not further used to evaluate the drug releasing performance. The LCST values obtained in PNIPAM-co-AAc derivatives were similar to previous study [37]. These results demonstrated that PNIPAM-co-AAc nanogels have two phase transitions and the LCST of PNIPAM containing AAc shifted to the higher temperature due to hydrophobic interactions from the interfacial PNIPAM chains and interelectronic repulsion via the carboxyl groups of AAc.

### Double Controlled Drug Release Performance

**Fig. 4:**
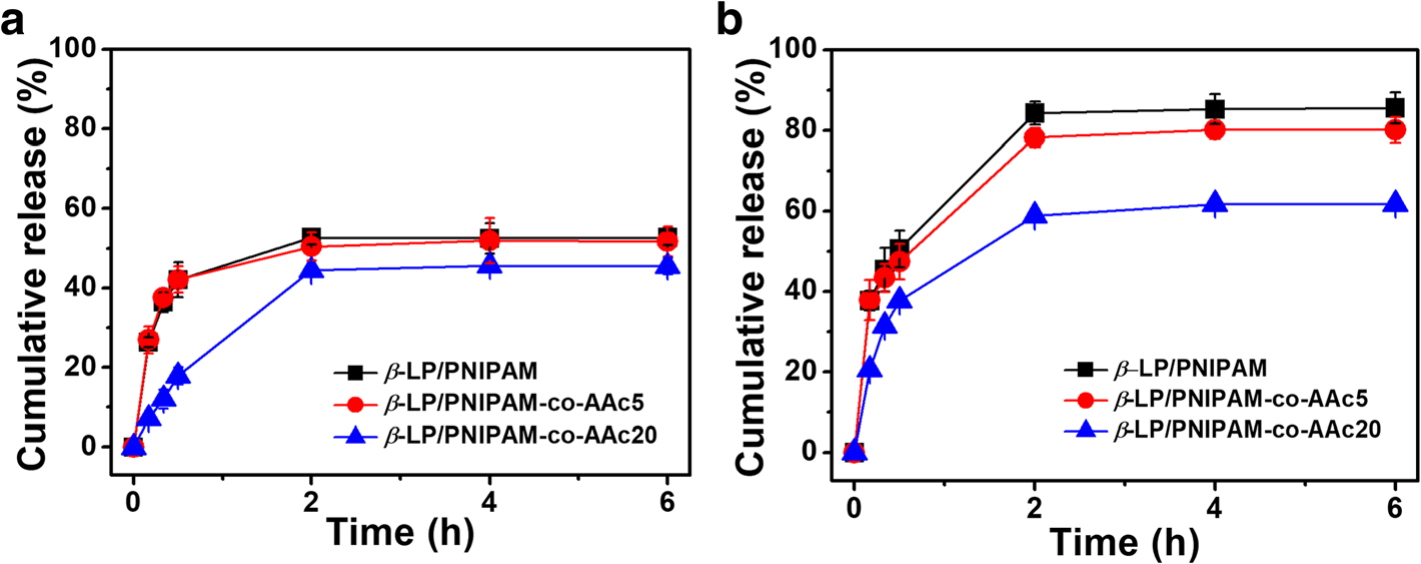
Cumulative release of β-LP from PNIPAM, PNIPAM-co-AAc5, and PNIPAM-co-AAc20 nanogels at temperatures of **a** room temperature (24 °C) and **b** body temperature (37 °C) and pH 7.4

To compare the drug release profiles of PNIPAM, PNIPAM-co-AAc5, and PNIPAM-co-AAc20, β-LP released from the PNIPAM-co-AAc derivatives was measured during a 6-h period at room temperature (24 °C) and body temperature (37 °C). Initially, we measured the UV–Vis absorption spectra of the PNIPAM-co-AAc20 and the PNIPAM-co-AAc20 including β-LP and observed a strong absorption at 257 nm corresponding to β-LP (Additional file 1: Figure S1). The drug loading capacity of PNIPAM-co-AAc20-loaded β-LP was found to be around 60% using a standard concentration-absorbance calibration curve of β-LP (Additional file 2: Figure S2) [38, 39]. As shown in Fig. 4, the cumulative percentage of drug released from PNIPAM-co-AAc derivatives showed that the amount of β-LP released from PNIPAM-co-AAc20 was relatively lower and its release efficacy was significantly reduced compared to PNIPAM and PNIPAM-co-AAc5 at both temperatures. However, the saturated drug release points of most PNIPAM-co-AAc derivatives were observed after the treatment within 2 h. In particular, the drug release efficiency of PNIPAM nanogels was highly affected by the reaction temperature. PNIPAM-co-AAc derivatives exhibited improved drug release efficiency at body temperature compared to that at room temperature. This result was also supported by the significantly higher cumulative drug release of all PNIPAM derivatives when the reaction temperature was over 40 °C (Additional file 3: Figure S3).

As shown in Fig. 4 and Table 1, PNIPAM-co-AAc nanogels at the high temperature could easily release the drug because of their remarkable shrinkage. Furthermore, the highest drug release efficiency at body temperature was observed in PNIPAM and the second highest efficiency was PNIPAM-co-AAc5. Both have a relatively low AAc content, leading to decreased LCST temperature. Especially, we observed that β-LP in PNIPAM-co-AAc20 was released with a relatively lower efficiency (61%) at body temperature, while in the other nanogels, approximately 80% of the β-LP was released at the same temperature. These results indicated that PNIPAM-co-AAc20 showed a minimal release of the drug at body temperature while encapsulating as much as possible, compared with PNIPAM and other PNIPAM-co-AAc5. Furthermore, these results were also consistent with temperature-dependent changes in the size measurement of PNIPAM derivatives to determine LCST values.

**Table 1:** LCST and release efficiency of PNIPAM derivatives at 24 °C and 37 °C

Hydrogels	LCST (°C)	Release efficacy (%)	
		24 °C	37 °C
β-LP/PNIPAM	32	53	85
β-LP/PNIPAM-co-AAc5	37	48	79
β-LP/PNIPAM-co-AAc20	45	46	61

**Fig. 5:**
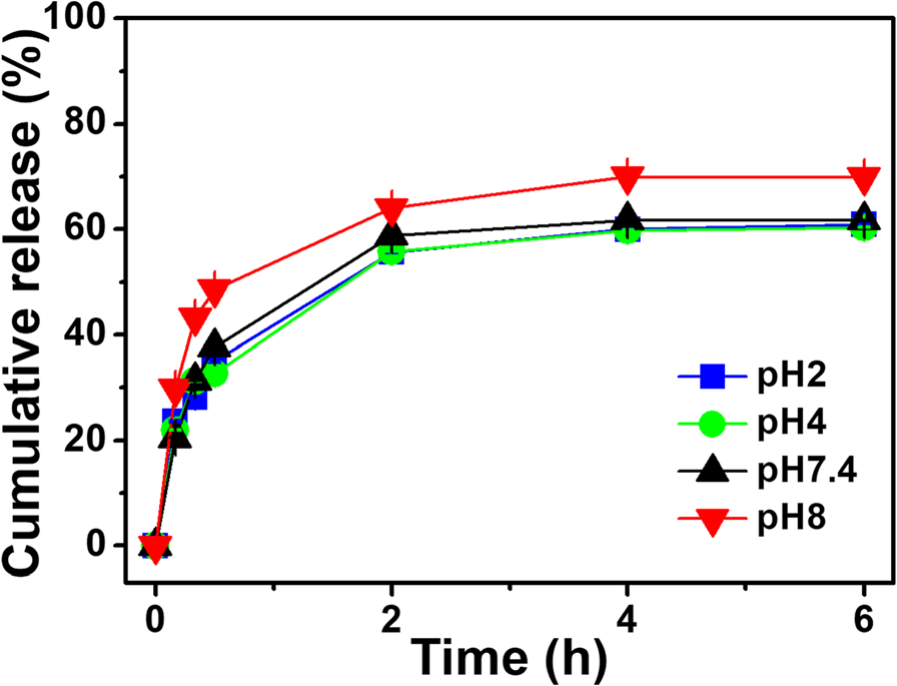
Cumulative release of β-LP from PNIPAM-co-AAc20 nanogels at various pH values

Next, we evaluated whether PNIPAM-co-AAc20 could control drug release via another factor to which PNIPAM responds, pH, with maximum entrapment of the drug at body temperature. PNIPAM-co-AAc20 showed approximately 70% cumulative maximum release efficiency, increasing by about 10% at pH 8 compared to either acidic or neutral pH. Meanwhile, no significant difference was observed between pH 7.4 and acidic pH (Fig. 5 and Table 2). Taken together, these findings indicate that the drug release profile of PNIPAM-co-AAc20 can be affected by controlling the content of AAc, and this double controlled drug release nanogel could effectively modulate the drug release rate at basic pH values which are known to be present in parts of the small intestines [40].

**Table 2:** Release efficiency of PNIPAM derivatives at various pH values

pH	Release efficacy (%)
2	60
4	60
7.4	61
8	69

### Evaluation of Drug Releasing Properties

**Fig. 6:**
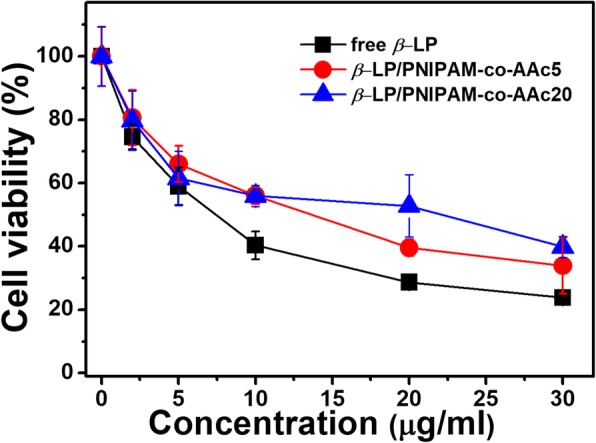
Anti-proliferative activity of PNIPAM-co-AAc nanogels loaded with β-LP at various concentrations in NIH3T3 fibroblast cells for 3 h at 37 °C

**Fig. 7:**
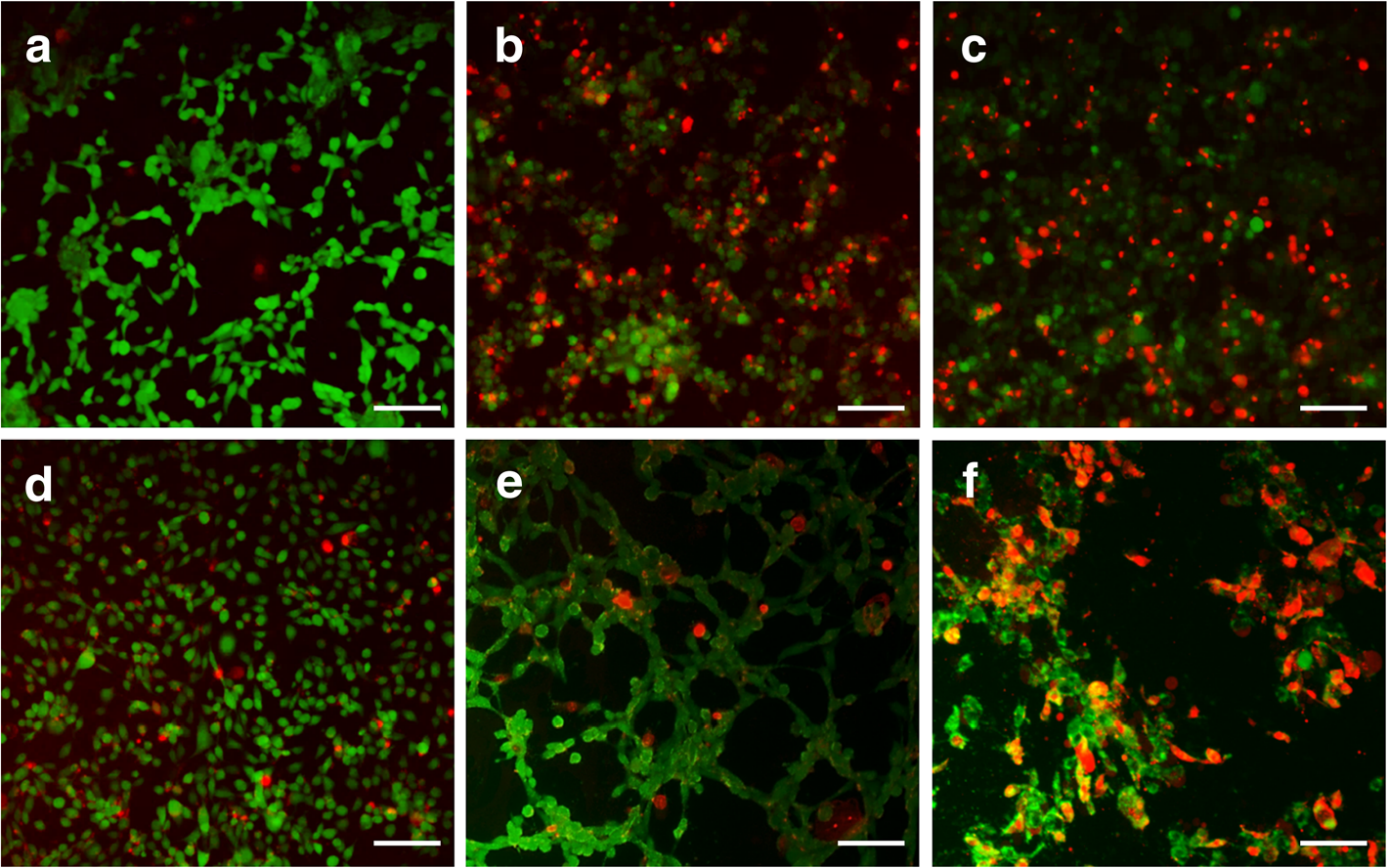
Fluorescent images of cytotoxicity in NIH3T3 cells with **a** untreated, **b** only β-LP, **c** β-LP/PNIPAM-co-AAc5, and **d** β-LP/PNIPAM-co-AAc20 treatment for 3 h at pH 7.4, and β-LP/PNIPAM-co-AAc20 treatment for 3 h (**e**) and 6 h (**f**) at pH 8.0. The live and dead cells are stained with calcein AM (green) and ethidium homodimer (red). Scale bars are 100 μm

In vitro anti-proliferation was evaluated to perform a key criterion of nanomaterials designed for controlled drug delivery and release. As indicated in Fig. 6, free β-LP showed lower cell viability than PNIPAM-co-AAc nanogels loaded with β-LP for equivalent concentrations of β-LP. Moreover, PNIPAM-co-AAc20 nanogel presented relatively high cell viability at a concentration of 20 μg/mL, because the β-LP release of PNIPAM-co-AAc20 nanogel was relatively low compared to that of the PNIPAM-co-AAc5 nanogel at 37 °C. In addition, this result also coincided with the cumulative drug release profiles. Then, we assessed the cell viability using fluorescently stained live and dead cells (Fig. 7). The live/dead cell staining assay showed that β-LP and PNIPAM-co-AAc5 nanogel including β-LP were similar in cell viability, while PNIPAM-co-AAc20 showed a significant increase in cell viability with a dose of 20 μg/mL after treatment for 3 h. However, enhanced drug release from PNIPAM-co-AAc20 started to be observed after incubation at pH 8.0 for 3 h and a significant, synergistic anti-tumor activity was seen at the same pH during the 6-h post-treatment. These findings implied that the temperature and pH dual responsive PNIPAM-co-AAc20 nanogel has a potential application for controlled drug loading and release in the terminal small intestine.

## Conclusions

We developed β-LP-loaded PNIPAM-co-AAc nanogels whose drug release can be triggered by temperature and pH. These nanogel derivatives were designed and prepared by radical co-polymerization. The LCST was raised with increasing AAc content of the PNIPAM-co-AAc nanogels because of interelectronic repulsion between the carboxylic groups on the AAc contents, resulting in the shrinkage of PNIPAM-nanogels and consequent drug release. PNIPAM-co-AAc nanogels with high AAc contents loaded with β-LP exhibited a markedly reduced in vitro release profile at body temperature. In addition, the drug release can be achieved with remarkable synergistic effect at basic pH. Finally, we demonstrate that PNIPAM-co-AAc20 has optimal properties, having reduced drug release efficiency at body temperature but enhanced drug release at pH 8.0, which is supported by cell viability assays using fibroblast cells. Therefore, this temperature- and pH-responsive nanogel could encourage a promising application for double controlled drug release at the physiological pH of the small intestines and an attractive modality for intestine targeted drug delivery via oral drug administration.

## Additional files

Additional file 1: **Figure S1.** UV-Vis absorption spectra for PNIPAM-co-AAc20 nanogels (Red) and PNIPAM-co-AAc20 nanogels loaded with β-LP (Black). (TIF 493 kb)

Additional file 2: **Figure S2.** Standard calibration curve of β-LP of PNIPAM-co-AAc20 at 257 nm to determine the loading efficiency. (TIF 481 kb)

Additional file 3: **Figure S3.** Cumulative release of β-LP from PNIPAM, PNIPAM-co-AAc5 and PNIPAM-co-AAc20 hydrogels at 46 °C. (TIF 483 kb)

## Abbreviations

AAc: Acrylic acid
DLS: Dynamic light scattering
FE-SEM: Field emission scanning electron microscopy
FT-IR: Fourier-transform infrared spectrometer
KPS: Potassium persulfate
LCST: Lower critical solution temperature
MBA: *N*,*N*′-methylenebisacrylamide
PNIPAM: Poly-*N*-isopropyl acrylamide
TEM: Transmission electron microscopy
β-LP: β-lapachone
