# Supplementary figures and images for: Dual Stimuli-Triggered Nanogels in Response to Temperature and pH Changes for Controlled Drug Release
Source: Nanoscale Res Lett. 2019 Mar 4;14:77. doi: 10.1186/s11671-019-2909-y (PMC6399374; doi:10.1186/s11671-019-2909-y)

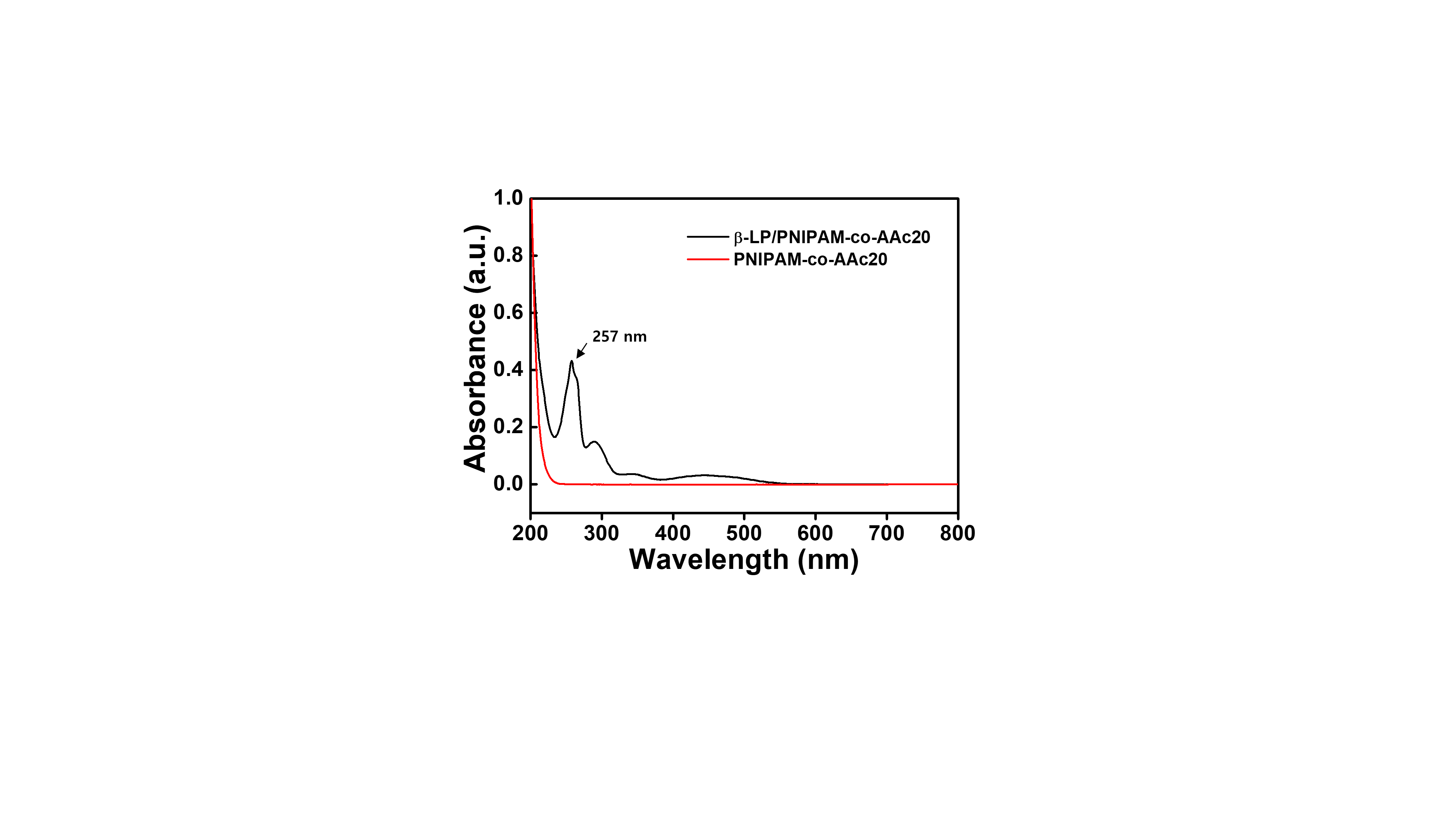

Supplement: Supplementary file 1 — Figure S1. UV-Vis absorption spectra for PNIPAM-co-AAc20 nanogels (Red) and PNIPAM-co-AAc20 nanogels loaded with β-LP (Black). (TIF 493 kb) [file 11671_2019_2909_MOESM1_ESM.tif]

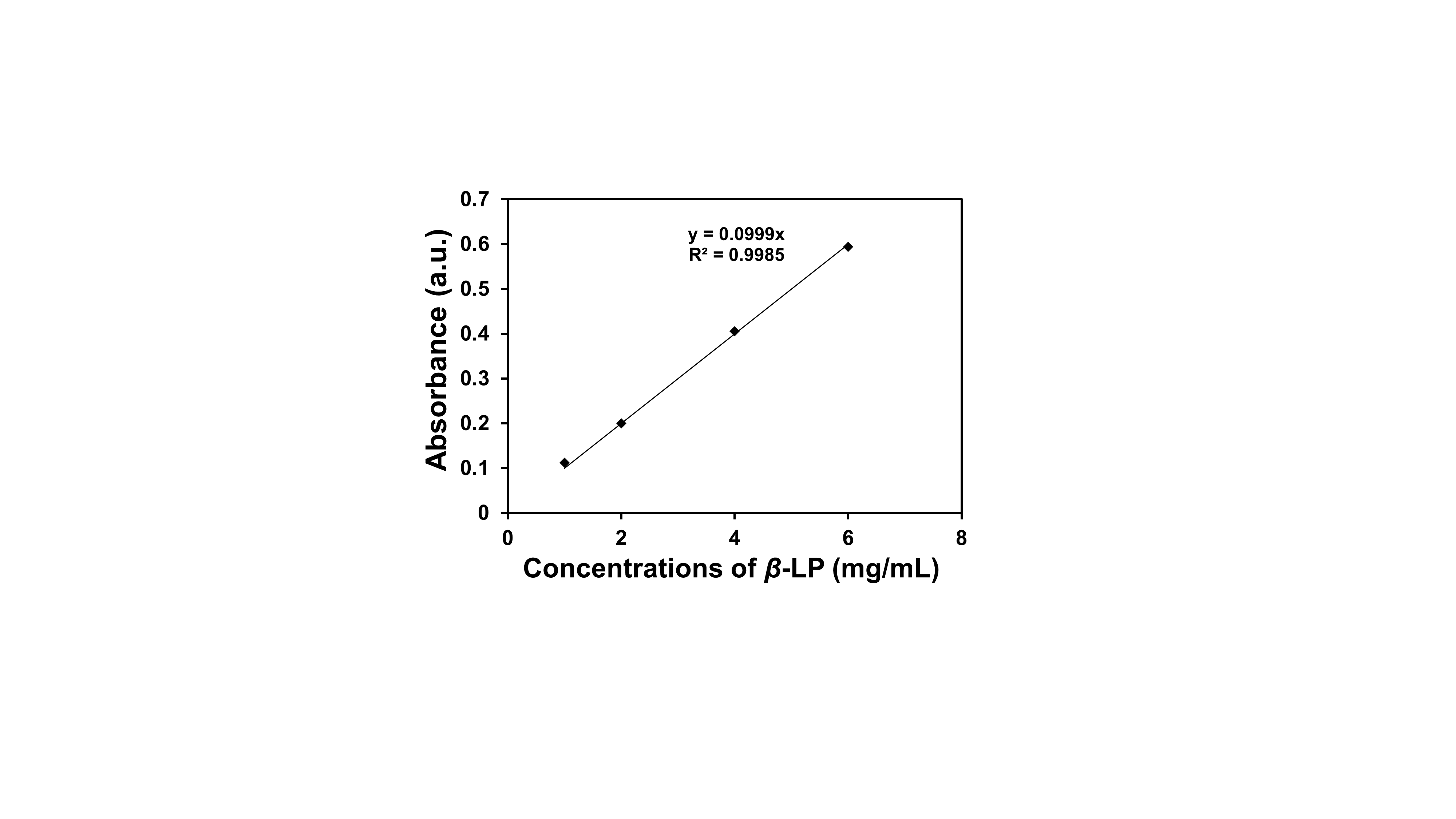

Supplement: Supplementary file 2 — Figure S2. Standard calibration curve of β-LP of PNIPAM-co-AAc20 at 257 nm to determine the loading efficiency. (TIF 481 kb) [file 11671_2019_2909_MOESM2_ESM.tif]

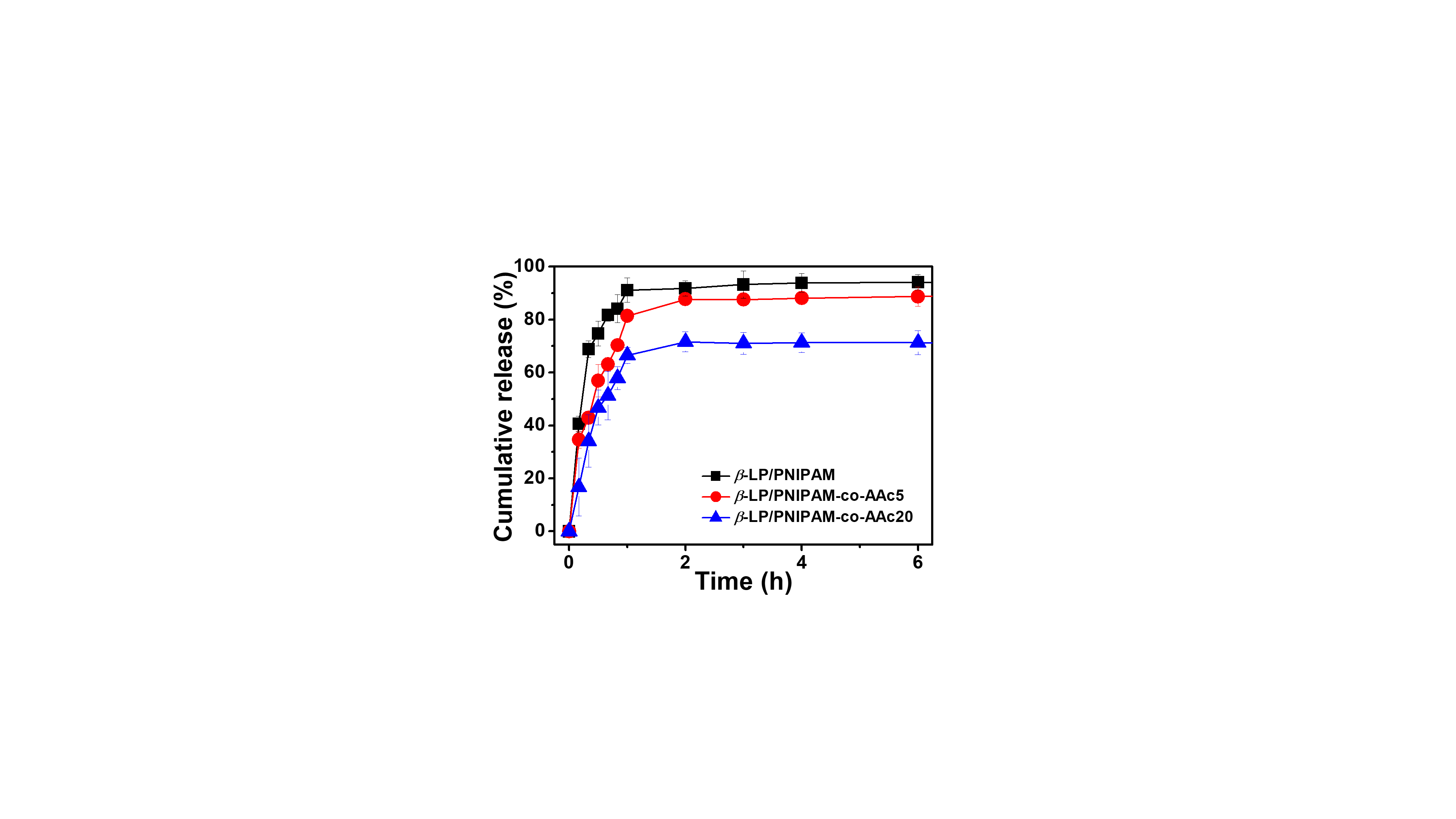

Supplement: Supplementary file 3 — Figure S3. Cumulative release of β-LP from PNIPAM, PNIPAM-co-AAc5 and PNIPAM-co-AAc20 hydrogels at 46 °C. (TIF 483 kb) [file 11671_2019_2909_MOESM3_ESM.tif]
